# Supplementary material for: Persister Cell Formation and Elevated lsrA and lsrC Gene Expression upon Hydrogen Peroxide Exposure in a Periodontal Pathogen Aggregatibacter actinomycetemcomitans
Source: Microorganisms. 2023 May 26;11(6):1402. doi: 10.3390/microorganisms11061402 (PMC10301654; doi:10.3390/microorganisms11061402)
Supplement: Supplementary file 1 [file microorganisms-11-01402-s001.zip › microorganisms-2344482-supplementary.pdf]

SUPPORTING INFORMATION

Persister cell formation and elevated *lsrA* and *lsrC* gene expression upon hydrogen peroxide exposure in a periodontal pathogen *Aggregatibacter actinomycetemcomitans*

Yohei Nakamura[1,2], Koji Watanabe[2], Yoshie Yoshioka[1], Wataru Ariyoshi[1], Ryota Yamasaki[1, 3*]

[1]Division of Infections and Molecular Biology, Department of Health Promotion, Kyushu Dental University, Kitakyushu, Fukuoka 803-8580 Japan
[2] Division of Developmental Stomatognathic Function Science, Department of Health Promotion, Kyushu Dental University, Kitakyushu, Fukuoka 803-8580 Japan
[3] Collaborative Research Centre for Green Materials on Environmental Technology,
Kyushu Institute of Technology, 1-1 Sensui-chou, Tobata-ku, Kitakyushu, Fukuoka 804-8550, Japan

*For correspondence. E-mail r18yamasaki@fa.kyu-dent.ac.jp

Tel. (+)81 93-285-3051

Keywords: *Aggregatibacter actinomycetemcomitans*; hydrogen peroxide; persister; periodontitis

Table S1. Number of cell divisions of the non-treated exponential *Aggregatibacter actinomycetemcomitans* cells and hydrogen peroxide-treated persister cells every half hour. % indicates the percentage of bacteria that dividing at that time.

	Exponential cells (Total=101 cells)		Persister cells (Total=4,291 cells)	
**Time (h)**	**Division**	**%**	**Resuscitation (division)**	**%**
**0**	0	0	0	0
**1.5**	31	30.69	5	0.12
**2**	61	60.40	2	0.05
**2.5**	5	4.95	4	0.09
**3**	1	0.99	5	0.12
**3.5**	0	0	8	0.19
**4**	0	0	7	0.16
**4.5**	0	0	6	0.14
**5**	0	0	8	0.19
**5.5**	0	0	3	0.07
**6**	0	0	1	0.02
**6.5**	0	0	0	0
**Total**	98	97.03	49	1.14

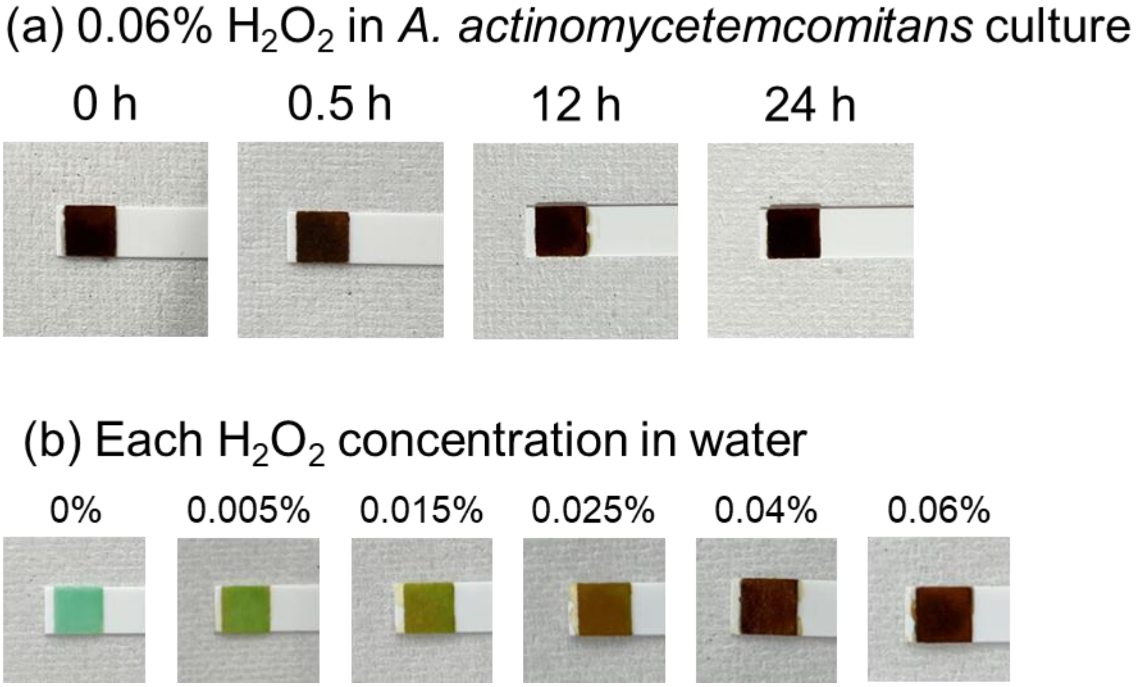

Figure S1. Hydrogen peroxide concentration test using test paper (Serim Research). (a) Testing the hydrogen peroxide concentration in the experiment in Figure 2b. No decrease in hydrogen peroxide concentration during 0–24 h in *A. actinomycetemcomitans* culture during hydrogen peroxide treatment. (b) Testing of hydrogen peroxide solutions added to water at various concentrations (test paper analysis).

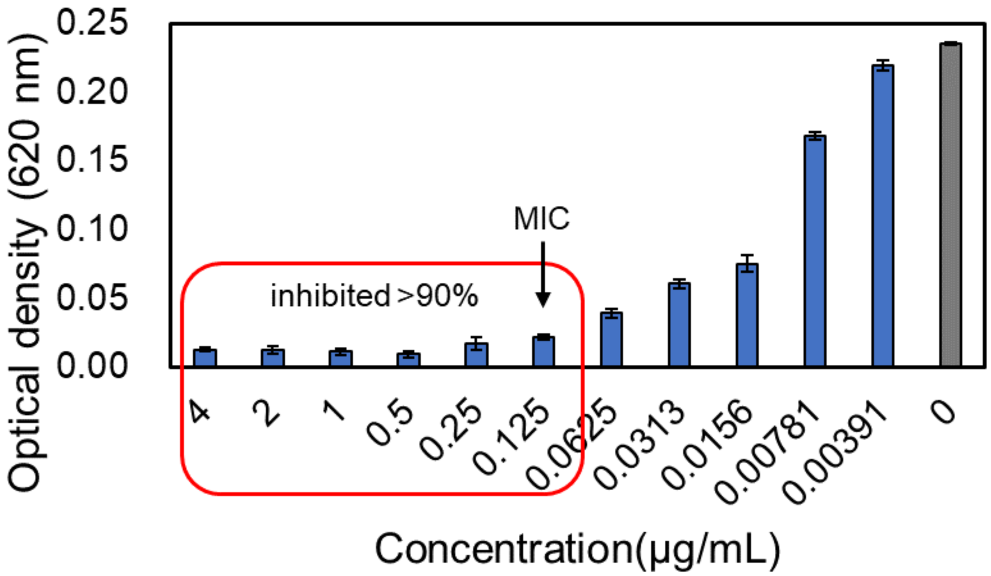

Figure S2. Growth inhibitory effects of mitomycin C (MMC) against *A. actinomycetemcomitans*. MMC value above 0.125 µg/mL inhibited more than 90 % of the bacterium. Hence, the MIC was defined at 0.125 µg/mL. This experiment was performed using three independent bacterial cultures.

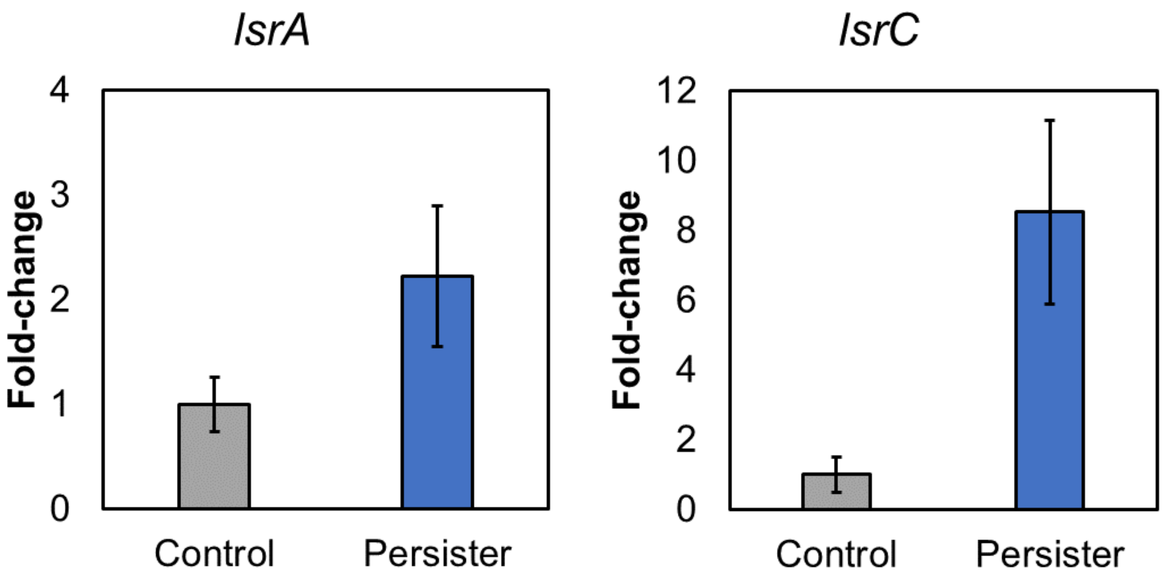

Figure S3. Quantification of *lsrA* and *lsrC* genes using real-time reverse transcription-polymerase chain reaction RT-PCR. Error bars indicate standard deviations of three independent samples.
